# Quantum Parametric Mode Sorting: Beating the Time-Frequency Filtering

**DOI:** 10.1038/s41598-017-06564-7

**Published:** 2017-07-26

**Authors:** Amin Shahverdi, Yong Meng Sua, Lubna Tumeh, Yu-Ping Huang

**Affiliations:** 1 Department of Physics and Engineering Physics, Stevens Institute of Technology, Hoboken, NJ 07030 USA; 2 Center for Distributed Quantum Computing, Stevens Institute of Technology, Hoboken, NJ 07030 USA; 3 Department of Electrical and Computer Engineering, Stevens Institute of Technology, Hoboken, NJ 07030 USA; 4 Department of Physics, Manhattan College, Riverdale, NY 10471 USA

## Abstract

Selective detection of signal over noise is essential to measurement and signal processing. Time-frequency filtering has been the standard approach for the optimal detection of non-stationary signals. However, there is a fundamental tradeoff between the signal detection efficiency and the amount of undesirable noise detected simultaneously, which restricts its uses under weak signal yet strong noise conditions. Here, we demonstrate quantum parametric mode sorting based on nonlinear optics at the edge of phase matching to improve the tradeoff. By tailoring the nonlinear process in a commercial lithium-niobate waveguide through optical arbitrary waveform generation, we demonstrate highly selective detection of picosecond signals overlapping temporally and spectrally but in orthogonal time-frequency modes as well as against broadband noise, with performance well exceeding the theoretical limit of the optimized time-frequency filtering. We also verify that our device does not introduce any significant quantum noise to the detected signal and demonstrate faithful detection of pico-second single photons. Together, these results point to unexplored opportunities in measurement and signal processing under challenging conditions, such as photon-starving quantum applications.

## Introduction

Time-frequency (TF) filters are the optimum linear systems for signal detection over interfering noise, such as those overlapping in both spectral and time domain^1–3^. For the measurement and processing of non-stationary signals, in particular, it is a key tool widely employed to attain high signal-to-noise ratio (SNR). There is, however, a fundamental tradeoff between the selection efficiency (i.e., the signal transmittance through the filter) and the noise rejection. On the one hand, to admit the signal with a higher efficiency, a wider TF filtering window is needed which unfortunately will also let through more noise. On the other hand, a tight window can reject most of the noise but at the price of a higher signal loss.

**Figure 1:**
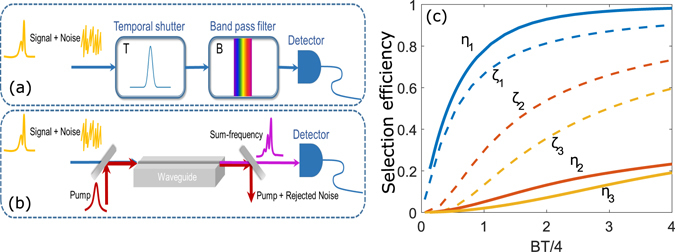
TF filtering versus QPMS. (**a**) and (**b**): Simplified schematics for a typical TF filter and a waveguide-based QPMS system, respectively; (**c**) The selection efficiencies of the first three signal modes {*ζ*_*i*_} for the TF filter in (**a**) and {*η*_*i*_} for QPMS system in (**b**), as a function of the time-bandwidth product.

**Figure 2:**
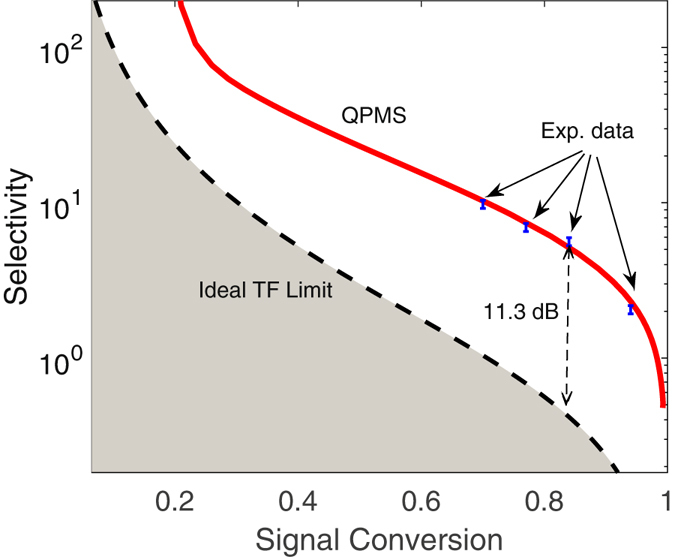
Selectivity versus signal selection efficiency. The dashed line plots the upper bound of ideal Gaussian TF filters, while the solid line shows the simulation results for a QPMS system with a Gaussian pump. The dots show our experimental result, as described in Section 2.6.

To illustrate this, we consider a typical TF filter consisting of a temporal shutter with a Gaussian profile followed by a Gaussian-shape bandpass filter, as shown in Fig. 1(a), whose full-width half-maximum (FWHM) are *T* and *B*, respectively. By the standard analysis^2–4^, we compute the filter’s normal modes (i.e., eigenmodes of its transform function between input and output) and their selection efficiencies (the eigenvalues), {*ζ*_*i*_}, as a function of the filter’s time-bandwidth product *BT*/4. This is numerically done by discretizing the transform function and then performing single-value decomposition^4–6^. Arranging the modes by descending efficiency, the best performance the filter can theoretically achieve is when the detected signal is in the first normal mode. In Fig. 1(c), we plot *ζ*_*i*_ for the first three modes. As seen, while a larger time-bandwidth product helps transmit the signal, the selection efficiencies for the rest modes increase, too, even more significantly. When the selection efficiency of the signal in the first mode reaches 90%, the efficiency for the second and third normal modes reaches 70% and 60%, respectively. As a result, a high signal selection efficiency comes necessarily at the price of high noise. To quantify this tradeoff, in Fig. 2 we plot the signal selectivity *S* versus the selection efficiency, where *S* is defined as *S* = *ζ*_1_/∑*ζ*_*j*_, for *j* > 1. In practice, *S* amounts to the SNR when the signal in the first mode is accompanied by broadband noise spanning many other orthogonal modes, with each’s occupation equal to the signal. As seen in Fig. 2, for a signal selection efficiency of 80%, the selectivity is −2.5 dB. We note that this tradeoff behavior does not pertain to the specific profile choices of the time shutter and bandpass filter, but similarly applies to all TF settings^4^.

The above tradeoff between the signal selection efficiency and noise rejection fundamentally limits the capabilities in measuring and processing weak signals buried in strong background noise, which represents a major challenge facing modern applications in photonics and beyond. An example is the quantum key distribution in free space, where the receiving of single signal photons can be hampered by strong atmospheric scattering and background photons spanning the same spectrum and arrival time^7–9^. Another is infrared wavelength detection, imaging and remote sensing, where the returning signal is swamped with blackbody radiated photons^10–12^.

Here we demonstrate an approach based on nonlinear wave mixing for improving the tradeoff inherent with the TF filters, thereby enabling faithful measurement and processing of weak signals even in the presence of strong interfering noise (e.g., those overlapping in both frequency and time). Specifically, we consider the parametric frequency conversion in nonlinear optical media that preserves the signals’ quantum states^13–16^, including their entanglement with other parties. We thus call it “quantum parametric mode sorting” (QPMS). The approach itself is nevertheless generally applicable to a breadth of devices and systems covering extended electromagnetic spectra^15, 17, 18^. The mode selectivity is achieved through engineering the normal mode structure of the frequency conversion process^5, 6, 19, 20^. While previous research has found its promises in quantum information applications using high-dimensional photons (see ref. 21 and references therein), here we demonstrate for the first time its potential for overcoming the SNR boundary with the TF filtering where the noise is randomly distributed in widespread, unknown modes. To be able to select the signal in an arbitrary time-frequency mode, we utilize the recently developed technique of optical arbitrary waveform generation (OAWG)^22, 23^.

Our approach exploits ultrafast nonlinear optics at the edge of phase matching. For frequency conversion, it is custom to use optical waves satisfying phase matching. However, by driving the frequency conversion with ultrafast pulses whose temporal width is shorter than the reciprocal of the phase matching bandwidth, interesting phenomena can occur where only photons in a single spatiotemporal mode are converted efficiently^6, 24^. The other modes, even spanning both the same spectrum and time, are not converted or converted with a much lower efficiency. This realizes QPMS whereby a signal is picked out of overlapping noises, similar to the TF filtering. Yet, thanks to coherent nonlinear effects, a significant advantage can be established over the TF filtering approach.

As a specific example, we consider a second-order nonlinear (*χ* ^(2)^) waveguide with phase matching bandwidth *B* driven by pump pulses in a Gaussian temporal profile with FWHM, *T*. The selection (i.e. conversion) efficiencies, {*η*_*i*_}, for the leading normal modes are calculated by normal-mode decomposition of the Green’s function describing the input-output relation of the waveguide^5, 25^ (which is numerically carried out via single-value decomposition, as {*ζ*_*i*_} are computed). The results are plotted in Fig. 1(c) as a function of *BT*/4. As shown, comparing with the TF filtering, a higher efficiency for the first mode and lower efficiencies for the other modes are obtained simultaneously at all time. It means that for the same efficiency, a much stronger noise rejection can be achieved, and vice versa. To benchmark this, in Fig. 2 we plot the selectivity *S* (defined similarly as for the TF filtering in the presence of broadband noise) versus the signal selection efficiency *η*_1_, which reveals a substantially improved performance from the TF filtering approach. For example, for the same signal selection efficiency at 80%, the selectivity is 8 dB for the current QPMS while only −2.5 dB for the TF filter, marking an improvement over 10 dB.

In the above comparison, we have assumed the signals to coincide with the first normal mode of each system. Thus the results in Figs 1 and 2 represent the best possible performance for those systems. In practice, however, the signal may vary from system to system and time to time. Matching the first normal mode of a TF filter to an arbitrary signal mode poses a daunting, if not insurmountable, challenge, especially when the signal contains multiple power peaks in time or spectrum. In contrast, with QPMS one can indirectly tailor the mode profiles and structures conveniently by optimally modulating the pump pulses via OAWG^25^. Furtheremore, dynamic OAWG was recently demonstrated capable of updating the waveform in real time^26–28^. Such development paves the way to a range of high-speed applications in photonic communications, sensing, and so on.

The rest of this paper is structured as follows. In Sections 2.1 to 2.3, we briefly present the theory, experimental setup, and numerical optimization results for QPMS. In Section 2.4, we show the characterizations of the system at the low conversion efficiency regime. In Section 2.5, we present the experiment of sorting two overlapping modes, directly comparing its performance to the TF filters. In Section 2.6, we demonstrate extracting the signal from broadband background noise, verifying a significant advantage of the current QPMS. In Section 2.7, we present the quantum measurement results, confirming that the QPMS is suitable for quantum operations. Finally, we conclude in Section 3.

## Results

### Theory

The present QPMS is realized through nonlinear mixing of a pump wave, a signal wave, and their sum frequency (SF) in a *χ* ^(2)^ waveguide, whose dynamics is described by a set of coupled Heisenberg equations of motion^6, 19^. Under the undepleted pump approximation, the waveguide’s input-output relation can be expressed using a Green’s function^6, 25^. Then, by modal decomposition, the normal modes and their eigenvalues of the Green’s function can be calculated, with the eigenvalues representing the selection efficiency of each mode; see ref. 6 for detailed calculation procedures. By engineering the phase matching properties of the waveguide and/or modulating the pump pulses, the mode structure of the SF generation, e.g., the detailed profile and selection efficiency of each normal mode, can be manipulated^5, 20^. In this work, we use a lithium-niobate waveguide that is periodically poled to phase match the SF generation between two waves in the the telecom C-band (see Section 4.1 for more information). The wavelength detuning between the two telecom waves is small (8 nm in our experiment), so that the difference between their group velocities is negligible over the rather short waveguide (1 cm). Thus the current QPMS effectively operates in the so-called “single sideband velocity matched” regime^6, 20^, where high mode-selectivity is obtained when the pump’s spectrum exceeds the waveguide’s phase-matching bandwidth Δ, or equivalently, the pump’s temporal width is shorter than *τ*, where *τ* ~ 1/Δ is the walk-off between the pump and SF wave in the waveguide. Here, an OAWG is employed to generate custom-tailored pump pulses for efficiently detecting desirable signals against other interfering signals or broadband noise.

### Experimental setup

**Figure 3:**
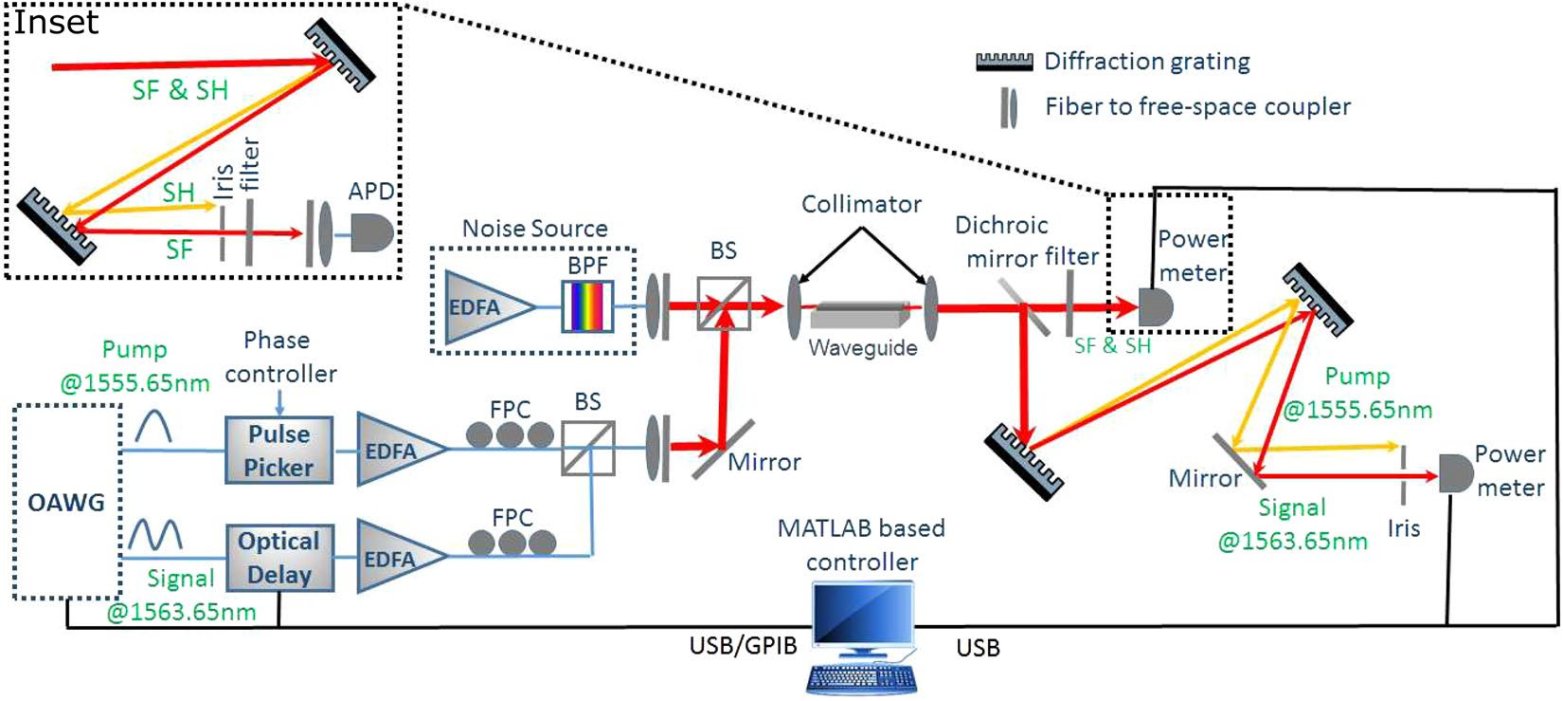
Experimental setup for QPMS. Pump and signal pulses are created via OAWG originally at 25 GHz repetition rate and downclocked using pulse pickers. The depleted signal and generated sum-frequency light are measured as a function of temporal delay between the input pump and signal. BPF: bandpass filter,  FPC: fiber polarization controller, BS: beam splitter, 3DTS: 3 dimensional translation stage, APD: Avalanche photodiode. The spectral filtering bandwidths are 1 nm and 0.8 nm at FWHM for SF and signal path, respectively.

The experimental setup is shown in Fig. 3, where two outputs of the OAWG are employed as the pump and signal pulse trains at 1555.65 nm and 1563.65 nm, respectively, which is the optimal choice given the constraints of the present OAWG and the waveguide’s phase matching (see Section 4.1). This 8 nm spacing limits the operable bandwidth of the pump and signal to ~800 GHz each, where we have access to 33 comb lines at 25 GHz spacing for the pump and signal, respectively. The pulse repetition rate of the pump can be reduced from 25 GHz down to 1 GHz by using a high-speed pulse picking system based on electro-optical modulation. The pump is amplified by an adjustable-gain erbium-doped fiber amplifier (EDFA) to achieve the required peak power (varying around 5 W) determined by our numerical simulation, (see Section 2.3), followed by a fiber polarization controller (FPC). The signal passes through a programmable optical delay line and an FPC, the former controlled by our automation software to scan the temporal delay between the pump and signal for measurement. The resulting pump and signal are combined using a 90:10 beam splitter and coupled into the waveguide. At the output, the pump and signal are first separated from the generated SF and undesirable second-harmonic (SH) waves by using a dichroic mirror. Then, the signal is separated from the pump using two grating filters and its power depletion is measured using an optical power meter. For the SF wave, a short-pass filter is used to additionally filter out the pump, before measured using another power meter. The optical delay line and both power meters are controlled by an automation software, so that the SF and the depleted signal power are measured simultaneously as the pump and signal are relatively delayed. For the former, the measured power also contains a small contribution from the weak but non-vanishing generated SH despite phase mismatching which is subtracted from the measured total power. The signal depletion due to the pump pulses are computed from the depleted average power based on the duty cycle of the pump.

**Figure 4:**
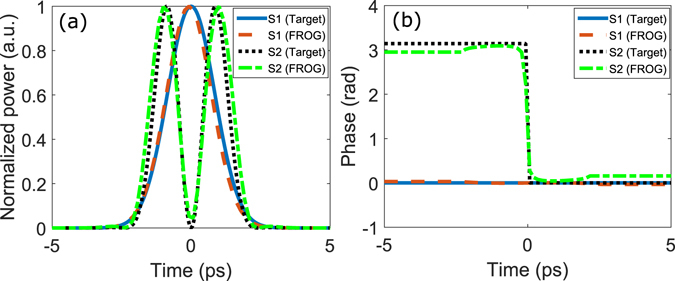
Retrieved pulse shapes. Two examples of the created pico-seconds pulses used in the experiment and retrieved pulse shapes by FROG, showing the normalized power (**a**) and phase profiles (**b**). The non-zero dip in (**a**) is likely due to the measurement noise of the FROG and the slight error in waveform retrieval. Note that retrieved phase profiles by FROG in (**b**) are valid when the normalized power is non-zero.

The shaped pump and signal pulses are generated through OAWG based on spectral line-by-line pulse shaping^22, 23, 29^. Here, the amplitudes and relative phases of 25-GHz evenly spaced frequency comb lines, generated by an optical frequency comb generator (WTEC-01-25), are each manipulated using a reconfigurable optical processor (Finisar 16000 A). Accessing to more than 100 comb lines, this system is capable of creating sub-picosecond pulses in arbitrary amplitude and phase profiles (see Section 4.2). The generated pulses are measured using a Frequency Resolved Optical Gating (FROG-HR 150, temporal resolution: 15 femtosecond) which characterizes the intensity and phase profiles of arbitrary pulses as short as 300 femtoseconds. Using these devices, we have been able to create arbitrary pulses with custom intensity and phase profiles with high fidelity. Two examples used in this experiment are shown in Fig. 4, where a good agreement between the designed profiles and measurement results is seen. The current OAWG can shape pulses with high fidelity at a temporal resolution of 400 femtosecond, although much wider pulses are used in our experiment restricted by the phase matching.

### Numerical optimization

We achieve selective conversion of an arbitrary mode by preparing the pump pulses in the optimized amplitude and phase profiles via OAWG according to the phase matching property of the waveguide. The optimization applies the standard random walk method to the phase and amplitude of each comb line^5, 30^. In this experiment, we use a 1-cm long periodic-poled lithium-niobate (PPLN) waveguide with negligible group velocity mismatch between the pump and signal. The inverse group velocity mismatch, i.e., the walk off, between the SF and the other waves is measured to be 235 *ps*.*m* ^−1^, and the internal conversion efficiency is 93% *W* ^−1^.*cm* ^−1^ (see Section 4.1).

**Figure 5:**
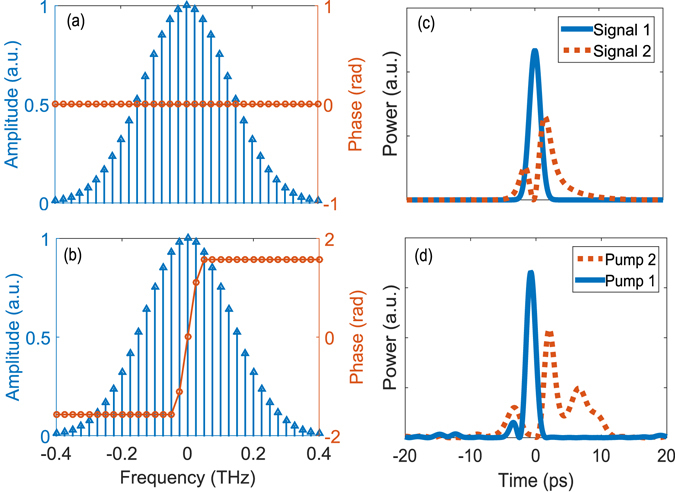
Distinguishing spectrally identical modes. (**a**) and (**b**) Spectral profiles of two input signal modes 100% overlapping in the spectrum (spectral width: 220 GHz), but with a *π*-phase flip across the spectral center. (**c**) Temporal profiles of the input signals, showing a temporal overlap >60%. (**d**) Temporal profiles of the optimized pump pules to selectively convert signal 1 and 2, respectively.

While our system can access a wide range of optical pulses, we consider specifically two cases of overlapping but orthogonal signal modes. In the first case, two input signal modes have an identical spectrum, i.e., they 100% overlap in the frequency distribution, but have a *π*-phase flip across the spectral center. Their spectral and time domain profiles are shown in Fig. 5, where the overlap in the time domain is greater than 60%. Here the overlap is defined as $|\int {{\rm{\Psi }}}_{1}{{\rm{\Psi }}}_{2}^{\ast }dt|$, where Ψ_1_ and Ψ_2_ are the normalized functions of the two modes. For each signal mode, a pump pulse is designed based on the numerical optimization to maximize its conversion while keeping the conversion of the other mode low. For the two signal modes in Fig. 5(c), the optimized Pump 1 and 2 are designed, respectively, as shown in Fig. 5(d). The conversion efficiency reaches 84% and 70% for the two modes, respectively, each with 12.5 dB and 8.3 dB selectivity. We note that this performance can be improved substantially by using a waveguide with a narrower phase matching band and/or more comb lines.

**Figure 6:**
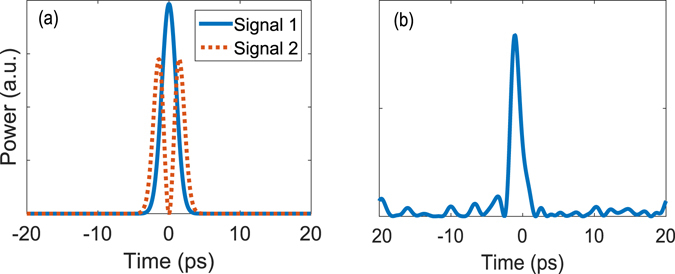
Distingushing TF filter’s modes. (**a**) shows the temporal profiles of the first two modes of a Guassian TF filter, where the pulse width of Signal 1 is 2 ps. (**b**) shows the pump pulse which is optimized to selectively convert Signal 1.

In the second case, Signal 1 and Signal 2 are chosen as the first two normal modes of the Gaussian TF filter with T = 5 ps and B = 500 GHz (both at FWHM), as shown in Fig. 6(a). For the TF filter, the selection efficiency is 84% for Signal 1 with a selectivity of 1.75 dB against Signal 2. For a direct comparison, we numerically design the pump pulse to achieve the same 84% conversion efficiency for Signal 1 while maximizing the selectivity over Signal 2. For the pump pulse profile in Fig. 6(b), our simulation shows an 8.45 dB selectivity, which corresponds to more than 6 dB improvement from the TF filter.

In the following sections, we first show the accuracy of our theory by verifying the system’s characteristics in the low conversion regime when the waveguide is weakly pumped. Then, we show that in the high conversion regime, the QPMS performance is significantly above the theoretical limit with the ideal TF filtering.

### Low-conversion regime

**Figure 7:**
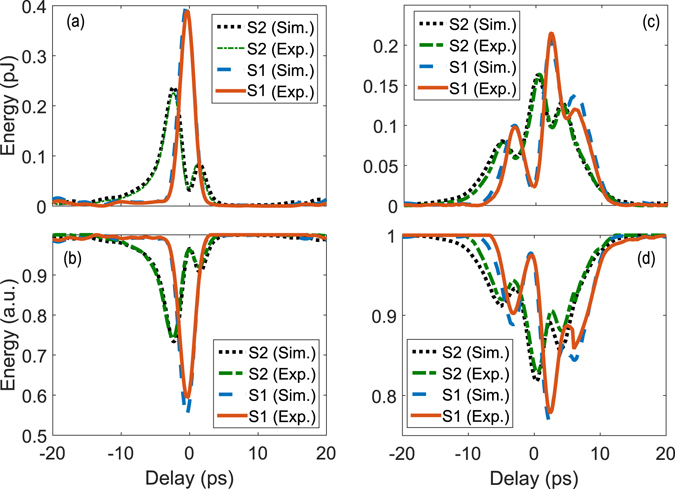
Low conversion regime measurement results. (**a**) and (**b**) show the measured and simulated SF and depleted-signal powers, respectively, for the two signals in Fig. 5 while they are relatively delayed from Pump 1 in Fig. 5. (**c**) and (**d**) plot the same but for Pump 2.

Here, we measure the generated SF between the pump and spectrally-identical signal pulses in the low conversion regime, as their relative delay is scanned. To fully capture the system’s characteristics, we monitor simultaneously the signal power depletion and the generated SF power, and compare both with our numerical results. Figure 7(a) and (b) show the measurement results when the pump, Pump 1, is designed to achieve about 40% conversion efficiency for Signal 1 in Fig. 5 while minimizing the conversion of Signal 2. As seen, the measurement is in an excellent agreement with the simulation without the use of any fitting parameter, which confirms our theory. In addition, the signal power depletion measurement coincides with the SF power measurement, both giving 40% conversion for Signal 1 and 4% for Signal 2. Next, we design another pump, Pump 2, to convert Signal 2 with about 20% efficiency while minimizing the conversion of Signal 1. The results are shown in Fig. 7(c) and (d), where both depletion and SF power measurement give 3% conversion efficiency for Signal 1. This result is on par with the result in ref. 31 for alike but different signal modes. While the agreement is still excellent between the experimental and non-fitting-parameter theoretical results when the pump and signals are aligned (i.e., zero delay), there are some deviations when the delay is large. One reason behind this might be that our simulation did not take into account higher-order group velocity dispersion effects, which may become significant for more complicated pulse shapes.

Table 1

**Table 1:** Conversion efficiency and selectivity in low conversion regime.

Spectral FWHM	Desirable mode					
	Mode 1			Mode 2		
	*Conv*_*exp*_ (%)	*S*_*exp*_ (dB)	*S*_*sim*_ (dB)	*Conv*_*exp*_ (%)	*S*_*exp*_ (dB)	*S*_*sim*_ (dB)
110 GHz	23 ± 1.15	9.17 ± 0.17	9.17	18 ± 0.90	8.53 ± 0.17	8.54
147 GHz	27 ± 1.35	8.45 ± 0.17	8.47	17 ± 0.85	7.11 ± 0.17	7.11
220 GHz	39 ± 1.95	9.87 ± 0.17	9.90	17 ± 0.85	7.59 ± 0.17	7.70

Measurement and simulation results for pulses 100% overlapping in spectral domain. *Conv*_*exp*_: Experimental Conversion Efficiency; *S*_*exp*_: Experimental Selectivity; *S*_*sim*_: Simulated Selectivity.

shows more results for different spectral widths (110, 147, and 220 GHz) for the signals and conversion efficiencies. All measurement results agree well with our simulation without the need for any fitting parameter. The selectivity for Signal 1 exceeds 8.45 dB for all spectral widths, and over 7 dB for Signal 2.

### High-conversion regime

To attain high conversion, the pump pulse train created via OAWG, originally at a 25 GHz repetition rate, is pulse picked to a 1 GHz rate in order to provide the required peak power as determined by the numerical simulation (on the order of Watts). The average power of the pump is monitored before coupling into the waveguide and controlled by the adjustable-gain EDFA, to obtain the desired peak power in the waveguide. In view of the exact coincidence between the signal power depletion and the generated SF power measurements seen in the low conversion regime, here we only monitor the latter to quantify the conversion efficiency.

**Figure 8:**
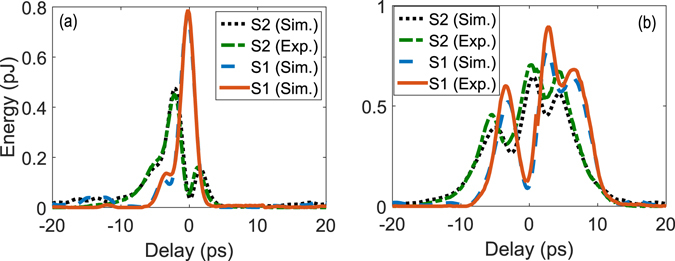
Selective conversion of spectrally identical pulses. The two signals are shown in Fig. 5. (**a**) compares the measured and simulated SF power for both signal modes when they are relatively delayed from the converting pump pulses whose phase and amplitude profiles are optimized for selective upconversion of Signal 1. (**b**) plots the same but with the pump optimized for Signal 2.

First, we consider the overlapping signals with identical spectrum. We design and create a pump, Pump 1, to attain a conversion efficiency of 84% for Signal 1 in Fig. 5 while minimizing the conversion of Signal 2. The measurement results are shown in Fig. 8(a) where a conversion efficiency of 84% and 6%, respectively, is measured for Signal 1 and Signal 2 when the pump and signals are temporally aligned. Again, a good agreement is seen between the simulation and measurement results without the using of any fitting parameter. Then, we design another pump, Pump 2, to convert Signal 2 with about 70% efficiency (limited by the pump peak power in our current setup) while minimizing the conversion of Signal 1. The results are shown in Fig. 8(b), where the conversion efficiency is measured to be 69% and 13% for Signal 2 and 1, respectively.

The superior performance of QPMS over the TF filtering occurs at the edge of the phase matching of the SF generation. To show this, we repeat the experiments for a series of signal modes in similar phase and amplitude profiles but of different spectral widths. The results are summarized in Table 2. With 84% conversion efficiency, the selectivity for Signal 1 exceeds 8.6 dB for a spectral width of 110 GHz (corresponding to 4 ps in pulse width for Signal 1) and increases to 11.3 dB as the spectral width doubles to 220 GHz. A similar trend is seen for Signal 2. In contrast, the best selectivity obtainable with the TF filtering technique is about 3 dB and 4.6 dB for the same conversion efficiency. These results confirm that a substantially higher selectivity for the same conversion efficiency is achievable with shorter pump and signal pulses relative to the reciprocal of the phase matching bandwidth, as theoretically shown in^6, 24^

**Table 2:** Conversion efficiency and selectivity in high conversion regime.

Spectral FWHM	Desirable mode							
	Mode 1				Mode 2			
	*Conv*_*exp*_ (%)	*S*_*exp*_ (dB)	*S*_*sim*_ (dB)	*S*_*TF*_ (dB)	*Conv*_*exp*_ (%)	*S*_*exp*_ (dB)	*S*_*sim*_ (dB)	*S*_*TF*_ (dB)
110 GHz	84 ± 2.52	8.63 ± 0.17	8.7	2.3	70 ± 2.10	6.50 ± 0.17	8	4.6
147 GHz	83 ± 2.49	8.75 ± 0.17	8.75	2.9	72 ± 2.16	6.77 ± 0.17	8.5	4.6
220 GHz	84 ± 2.52	11.3 ± 0.17	12.5	2.4	69 ± 2.07	7.18 ± 0.17	8.32	3.8

Measurement and simulation results for pulses 100% overlapping in spectral domain. *S*_*TF*_: Simulated Selectivity for the TF filter.

. In addition, a higher pulse shaping resolution can also help increase the mode selectivity. In all cases, the measurement results agree well with our simulation without the need for any fitting parameter. We note that our simulation has found that the selectivity of 16 dB and 12 dB can be achieved for Signal 1 and Signal 2, respectively, with a 440 GHz spectral width. However, the required pump pulse shape cannot be generated because of the bandwidth limitation with the current waveguide and OAWG device.

**Figure 9:**
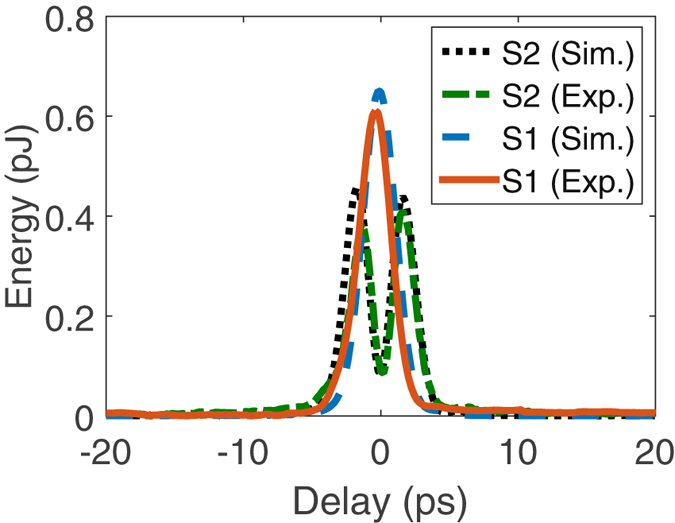
Selective conversion versus signal-pump delay. Measured and simulated SF power for the second example described in Section 2.3, where the signals correspond to the first two modes of a Gaussian TF filter, and the pump is optimized to selectively convert Signal 1 with a 2-ps pulse width.

Next, as another direct comparison with the TF filtering, we consider the first two normal modes of a Gaussian TF filter, with T = 5 ps and B = 500 GHz (both are FWHM), as shown in Fig. 6. We design and create a pump to achieve a conversion efficiency over 80% for the Signal 1 while minimizing the conversion of Signal 2. The measurement results are shown in Fig. 9 for Signal 1 (in equivalence with 220 GHz), where good agreement is again seen between the simulation and measurement results. The selectively is 8.5 dB, which represents a 6.8 dB improvement over the ideal TF filtering result. This selectivity is on par with the value (~9 dB) reported in ref. 14 for a much longer PPLN waveguide (~6 cm) while it shows >40% improvement compared with those in refs 17, 31 at almost the same level of conversion efficiency.

### Selectivity in the presence of broadband noise

The above results have shown the advantage of QPMS over the TF filtering for overlapping but known signal modes, whose applications can be found in areas of quantum information processing^21^. Another important application of QPMS is to reject background noise overlapping with the signal, which can spread evenly over a large number of modes. In Fig. 2, this noise rejection, quantified by the signal selectivity, is plotted versus the selection efficiency for both QPMS and the TF filtering techniques, where an infinite number of noise modes are considered and the photon occupation per noise mode is assumed to be the same as the signal. As suggested by the simulation results, a significant improvement in the selectivity is achieved for QPMS over the TF filtering in both low and high conversion regimes.

To verify this advantage, we use the same signal and pump shapes as Signal 1 and Pump 1 in Fig. 5, where the pump pulse train is down clocked to 1 GHz so as to obtain the required peak power at different levels of conversion efficiency. To simulate the background noise, we create a broadband noise utilizing the amplified spontaneous emission (ASE) of an adjustable gain EDFA, whose output is filtered by a wavelength division multiplexing (WDM) filter centered at the signal wavelength (FWHM bandwidth: 4 nm). The photon occupation of each mode is then computed from the total beam power, using the facts that the mode occupation is uniform within the WDM band and the ASE is unpolarized. The noise and signal are then combined at a 70:30 beam splitter, as shown in Fig. 3. For the same pump, the SF light power from the noise and the signal are measured individually. The selectivity is calculated by normalizing the photon occupation of the noise modes to match with the signal, which turns out to be 7.5 dB for an 84% selection efficiency. In contrast, the upper limit achievable with a Gaussian TF filtering is −3.8 dB for the same selection efficiency (84%). This amounts to an improvement from the theoretical limit of the TF filtering by 11.3 dB. As seen in Fig. 2, these measurement results lie right on top of the theoretical curve from our simulation without the use of any fitting parameter, which clearly validates our anticipations.

### Single photon level upconversion

To examine the utilities of our approach in areas of quantum optics, particularly those photon starving applications like long distance quantum key distribution over lossy free space^9, 32^, we next assess the intrinsic noise level and perform conversion of single photons.

In our system, background photons can be created in the SF band predominantly through three processes: (i) the frequency upconversion of the Raman photons emitted in the signal band (centered at 1564.65 nm) by the pump (centered at 1554.65 nm), (ii) the Raman scattering of the SH light created by the pump, (iii) the upconversion of the photons emitted by the SH light into the signal band via second-harmonic spontaneous parametric downconversion^33^. We count the total background photons in the SF band for a 6.8-ps pump pulse similar to the one shown in Fig. 5(d) by using a silicon Avalanche Photon Diode (APD) with an ultra-low dark count, e.g., 1.8 Hz in the free-running mode. The pump peak power of the current setup is limited to about 700 mW, which gives 40% conversion efficiency for a signal mode of identical shape as Signal 1 in Fig. 5(c) but twice longer. As shown in Fig. 3, the noise photons are collected using subsequential one bandpass (extinction ratio: 25 dB) and two single-grating filters (extinction ratio: 45 dB for each) with center wavelength at 779.83 nm and FWHM bandwidth of 1 nm, which provide together a filtering extinction ratio of over 115 dB. The number of modes detected is 3.7^13^. The total detection efficiency of our system is measured to be 2.7%, which includes the transmission loss of the filters, the free-space to fiber coupling loss, and the APD’s quantum efficiency.

The raw noise photon counting is 5.52 × 10^−7^ per pump pulse, which, after taking into account the total detection efficiency, amounts to (2.05 ± 0.0061) × 10^−5^ per pulse. This number can be further reduced to 0.55 × 10^−5^ per pulse by reducing the number of detected SF modes to one^13^. The rather low noise level is on par with the dark count probability of the best commercially available InGaAs APD’s for the same wavelength range. This indicates that the frequency conversion will preserve the quantum states of the photons, as established in previous studies of similar systems^34–37^. The noise is attributed to the discrete phonon modes of the lithium niobate crystals^38^ and relatively weak Raman scattering at a small detuning between the pump and signal^37, 39^. To further lower the noise level, one can either adjust the detuning^14^, or carefully engineer the waveguide’s phase matching to additionally suppress the parasitic SH generation of the pump. With the capability of distinguishing overlapping modes and an effective detection timing jitter defined by the pump (~ps or less), the photon detection based on this upconversion pave a path to new areas of operation in quantum optics, e.g. noise tolerant high dimensional quantum key distribution^21^.

**Figure 10:**
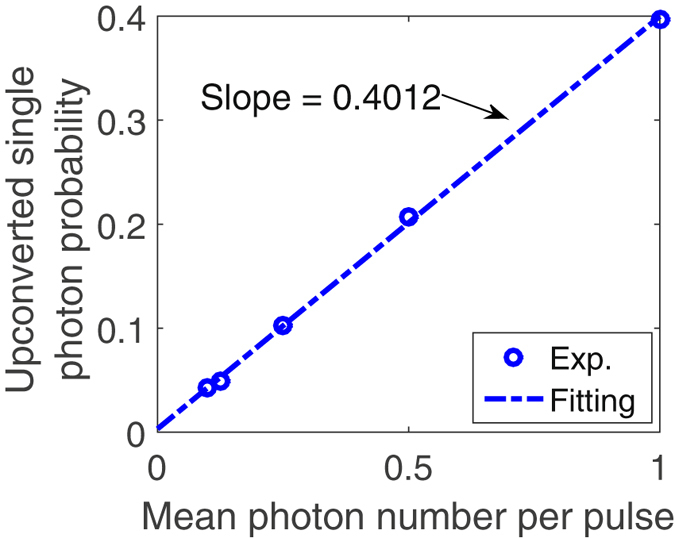
Single photon up-conversion. Blue dots show the probability of up-converted single photons versus mean photon number per pulse of the signal (spectral width: 110 GHz) while the dashed line shows the linear fitting.

To demonstrate the operation of the present QPMS at a single-photon level, we attenuate the signal to various photon number per pulse and count the up-converted SF photons. Figure 10 plots the SF photon counting, after accounting for the total detection efficiency, for mean photon numbers per pulse varying between 0.05 to 1 and a linear fit. The fitted slope gives a 40% internal conversion efficiency of the signal photons, which is in an excellent agreement with our simulation validated in the previous classical measurement.

## Conclusion

By exploiting the sum-frequency generation at the edge of phase matching in a commercial PPLN waveguide, we have demonstrated mode-selective detection of picosecond signals in the presence of overlapping modes and broadband noise. It utilizes an optical arbitrary waveform generator to optimally modulate–for the given phase matching property–the amplitude and phase profiles of the pump and signal pulses, each consisting of 33 frequency comb lines at 25 GHz spacing. Given the restricted optical bandwidths in the current setup, we have distinguished spectrally identical and temporally overlapping signals with high selectivity (~11 dB) and mode detection efficiency (84%), which represents a >6.5 dB improvement from the theoretical limit of a typical Gaussian TF filter. Furthermore, we have observed the superior performance of QPMS over the TF filtering in picking weak signal from broadband background noise, achieving a >11 dB improvement over the latter. We have also verified that the excessive noise introduced by our device can be made negligible and showed the faithful detection of single photons. Our results highlight a viable approach for overcoming the fundamental tradeoff with the TF filters between the signal selection efficiency and signal to noise, which may enable otherwise prohibited measurement and processing of weak quantum signal in the presence of strong interfering noise.

## Methods

### Waveguide characterization

**Figure 11:**
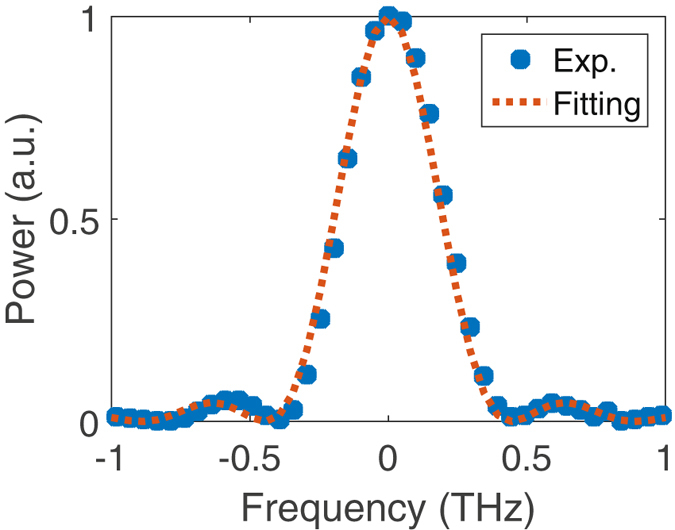
Phase matching profile of the PPLN waveguide. Blue dots show the experiment results at 40 °*C* and dashed line shows the *sinc* ^2^ fitting, plotted against the frequency offset of the SH wave from the phase matching peak (779.825 nm). The FWHM phase matching bandwidth is about 400 GHz centered at 779.825 nm, which is equivalent to 200 GHz for the lightwaves﻿ centered at 1559.65 nm.

The phase matching curve of the 1-cm long magnesium doped periodically poled lithium niobate (MgO:PPLN) waveguide is measured at 40 °*C*. A continuous wave laser is coupled (overal coupling efficiency is about 75%) with TE poarization into the waveguide and swept from 1554 nm to 1566 nm, while the SH power is measured. As shown in Fig. 11, the measurement data lie on top of a *sinc* ^2^ ideal phase matching profile, from which the inverse group velocity mismatch between SF and the other waves is calculated to be 235 *ps*.*m* ^−1^ for our numerical optimization. With the power levels in the current experiment, the self-phase modulation and photorefractive effects are negligible.

### Programmable OAWG

**Figure 12:**
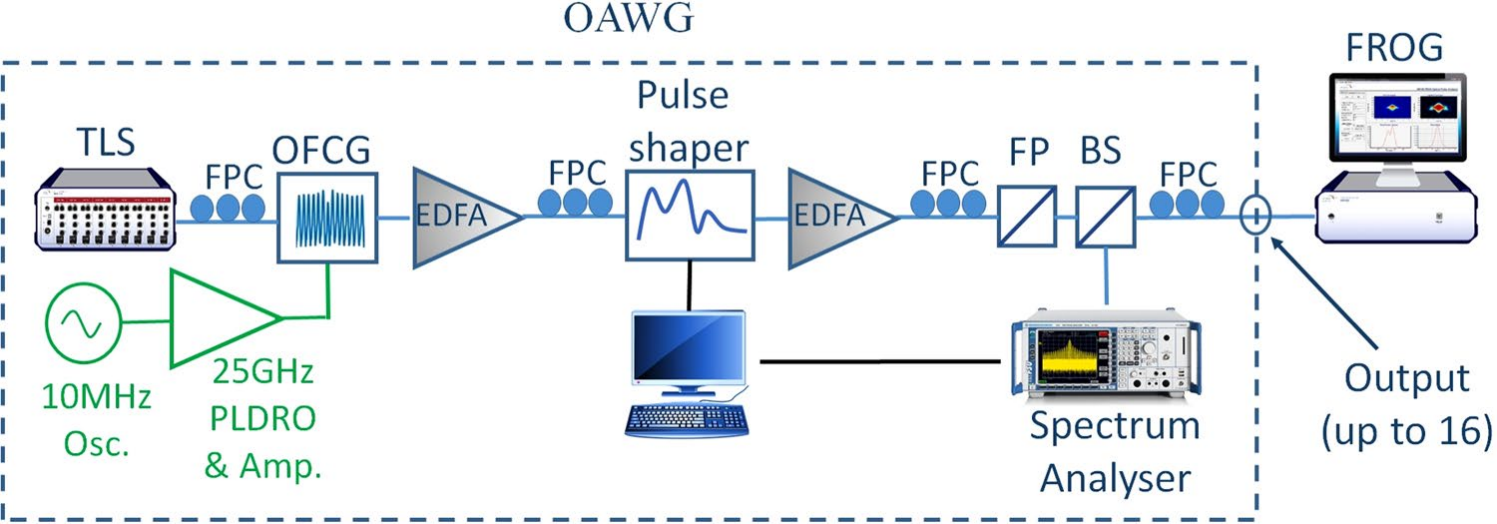
Schematic of the programmable OAWG. TLS: tunable laser source, OFCG: optical frequency comb generator, FPC: fiber polarization controller, FP: fiber polarizer, BS: beam spliter.

The OAWG system in Fig. 12, includes: a tunable laser, an optical frequency comb generator (OFCG WTEC-01-25), a reconfigurable optical processor (Finisar 16000 A), erbium doped fiber amplifiers (EDFA’s), an optical spectrum analyser (OSA) and a central controller for all parts. Based on spectral line-by-line pulse shaping, the intensities and relative phases of 25-GHz evenly spaced frequency comb lines are manipulated individually. This system is able to create arbitrary pulses with custom intensity and phase profiles with high fidelity as short as 300 femtoseconds.

### Data availability

The data that support the findings of this study are available from the corresponding author upon request.
